# Umbilical amnion and amniotic membrane transplantation for infectious scleritis and scleral melt: A case series

**DOI:** 10.1016/j.ajoc.2021.101013

**Published:** 2021-01-23

**Authors:** Zeba A. Syed, Christopher J. Rapuano

**Affiliations:** a Cornea Service, Wills Eye Hospital, Sidney Kimmel Medical College at Thomas Jefferson University, Philadelphia, PA, USA

**Keywords:** Amniotic membrane, Infectious scleritis, Melt, Sclera, Umbilical amnion

## Abstract

**Purpose:**

To report the outcomes of two cases of infectious scleritis with severe scleral thinning managed using combined mechanical debridement, cryopreserved umbilical amnion (UA) and amniotic membrane (AM) grafts, and antimicrobial therapy.

**Observations:**

Two patients presented with severe infectious scleritis with progressive scleral melts secondary to *Pseudomonas aeruginosa* subsequent to pterygium surgery and intravitreal injection, respectively. Surgical debridement was followed by UA grafting, AM transplantation, and antimicrobial therapy. Epithelialization and vascularization were seen within 1–4 weeks post-operatively. At last follow up of 5–6 months, the structural integrity of the sclera remained preserved with no infection recurrence, graft necrosis, or wound dehiscence.

**Conclusions and importance:**

This case series highlights the utilization of UA grafting along with AM transplantation and topical and systemic antibiotics to preserve the globe in cases of severe infectious scleritis.

## 1 Introduction

Infection is a serious but rare cause of scleritis, representing about 5–10% of all scleritis cases.^1^ Infectious scleritis (IS) is associated with longer resolution times and worse outcomes compared to non-infectious causes,^2^ and can result in a number of vision-threatening complications including cataracts, glaucoma, endophthalmitis, and loss of the globe.^3^^,^^4^ Topical and systemic antibiotic regimens alone are often inadequate, as the biofilm, avascularity of the sclera, and dense collagen network limit antibiotic penetration and reduce bacterial susceptibility.^5^^,^^6^ Thus, surgical debridement is often used to facilitate antibiotic access.^3^^,^^7^^,^^8^

A number of studies have reported favorable outcomes using debridement in conjunction with antibiotics for the treatment of IS.^4^^,^^5^^,^^7^ In cases with severe thinning, a patch graft is often used to help maintain structural integrity of the globe. Umbilical amnion (UA) and amniotic membrane (AM) grafts have been used extensively in ophthalmology,^9^^,^^10^ however, their use in the treatment of IS and for severe scleral thinning has not been reported. Herein, we describe two cases of IS that were successfully treated with mechanical debridement, UA grafting and AM transplantation, and antimicrobial therapy.

## 2 Findings

### 2.1 Case 1

**Fig. 1:**
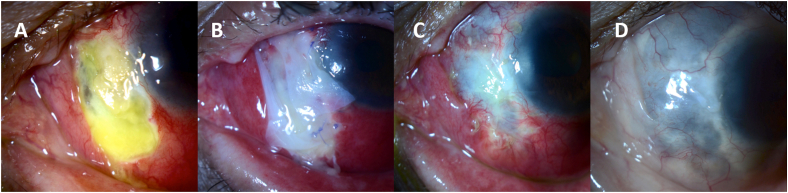
**Case 1.** Patient presented with 9 mm × 5 mm scleral melt (A) that was surgically managed using umbilical amnion patch grafting and amniotic membrane transplantation. Post-operative photos after 1 day (B), 1 month (C), and 3 months (D) are presented.

A 73-year-old man presented with persistent pain OS despite treatment with topical antibiotics for approximately 2 weeks. He had a history of pterygium surgery with mitomycin-C in this eye 5 years prior. The visual acuity was 20/200 and intraocular pressure (IOP) was 11 mmHg. Examination revealed 9 mm × 5 mm scleral necrosis nasally with purulent material and scleral plaques. There was significant scleral thinning with uveal show but no uveal prolapse (Fig. 1). There was also a temporal scleral plaque without active scleral necrosis. The patient had inferior keratic precipitates (KPs) and 2+ nuclear sclerosis (NS). The diagnosis was confirmed to be infectious scleritis with cultures growing *Pseudomonas aeruginosa*.

The patient was taken to the operating room. Toothed forceps and a 57-blade were used to carefully dissect the nasal scleral plaque, for which pathologic evaluation demonstrated dense calcification. The necrotic scleral material was then debrided with a 57-blade and sent for additional cultures. Dilute ceftriaxone was used to wash out the area of necrotic sclera. A 0.75 cm × 1.0 cm UA patch graft (AmnioGuard; Bio-Tissue, Miami, FL) was placed over the area of scleral thinning and secured into place using 16 interrupted 7-0 Vicryl sutures. The graft was trimmed at the edges so that it was flush with the edge of the corneal limbus. Next, a 2.5 cm × 2.0 cm AM (AmnioGraft; Bio-Tissue, Miami, FL) was cut in half. The first half was placed over the UA patch graft, and was secured in place with Tisseel fibrin glue (Baxter, Deerfield, IL). The other half was placed over the first AM and secured with 4 interrupted 7-0 Vicryl sutures at the corners of the graft. Subconjunctival aliquots of ceftriaxone were administered surrounding the area of infection, and the eye was patched and shielded. Post-operative drop regimen included fortified tobramycin 14 mg/ml and topical moxifloxacin 0.5% every 2 h with a taper over six weeks, oral ciprofloxacin 500 mg twice daily for four weeks, and cyclopentolate 1% twice daily for six weeks. Topical prednisolone acetate 1% four times daily was started three weeks after surgery and was tapered off over the ensuing 10 weeks.

On post-operative day 1, the patient's vision was 20/300. The UA with overlying AM remained in good position. Slit lamp examination revealed 3+ conjunctival injection, inferior KPs, a small hyphema, and 2+ NS. At 1 week, the visual acuity was counting fingers due to worsening of the cataract, and IOP was 8 mmHg. Examination revealed that the UA remained in good position with staining inferiorly and dissolution of the overlying AMs. There was 3+ conjunctival injection diffusely, a chemotic plica, an area of depression between the plica and border of the AM graft, inferior KPs, a sliver hyphema, and 2–3+ brunescent NS. By 1 month, the visual acuity remained at counting fingers, and IOP was 10 mmHg. Examination revealed similar findings as 1 week, however the UA showed early vascularization with no staining present. The conjunctival inflammation had decreased to 2+ injection and hyphema had resolved. At 3 months, visual acuity had slightly improved to 20/400, and IOP was 12 mmHg. Examination revealed that the UA had dissolved with residual vascularization and no staining, a quiet conjunctiva, cornea with no infiltrate or epithelial defect, and 3+ brunescent NS with pigment on the anterior capsule (Fig. 1). Six months post-operatively, the patient underwent cataract extraction with intraocular lens placement. His 1-week post-operative visual acuity was 20/40.

### 2.2 Case 2

**Fig. 2:**
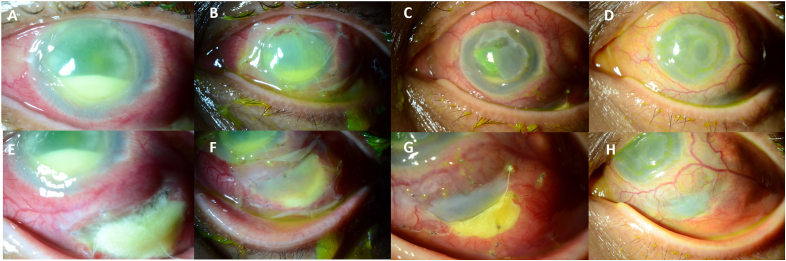
**Case 2.** Patient presented with a 4 mm × 6.5 mm inferotemporal scleral abscess, a 9 mm × 10 mm corneal epithelial defect, and a 3.5 mm hypopyon (A, E). Surgical intervention resulted in progressive healing and epithelialization at 1 day (B, F) and 1 week (C, G), with complete epithelialization at 8 weeks (D, H).

A 64-year-old female was referred to the Wills Eye Hospital Emergency Room for a culture-positive infectious corneal ulcer secondary to *Pseudomonas aeruginosa* that had failed topical antibiotics and had progressed to suspected endophthalmitis. The patient underwent an intravitreal tap and injection for presumed keratitis-related endophthalmitis, and subsequently developed scleritis two weeks later in the location of the intravitreal injection, at which point she was referred to our practice. The visual acuity was hand motion and IOP was 4 mmHg. Slit lamp examination was notable for a 4 mm × 6.5 mm inferotemporal scleral abscess that was non-contiguous with the cornea, a diffusely edematous corneal stroma with patchy tissue loss (ranging from 40 to 80%), a 9 mm × 10 mm corneal epithelial defect, and a 3.5 mm hypopyon (Fig. 2). The patient was taken to the operating room for corneal biopsy and a single-layer AM graft covering the involved sclera, with injection of subconjunctival cefazolin 50 mg/ml and gentamicin 40 mg/ml. Despite this intervention and inpatient treatment with intravenous levofloxacin 750 mg daily and hourly topical fortified tobramycin 14 mg/ml and topical moxifloxacin 0.5% for seven days, the patient had progressive scleral melt and was taken to the operating room for more definitive surgery.

The surgery was performed similar to that described for Case 1. The necrotic inferotemporal scleral material was resected and sent for pathology evaluation. The area of scleral necrosis was measured to be 5 mm × 9 mm, and a UA patch graft was cut to size and secured with interrupted 9-0 Vicryl sutures. A 8 mm × 12 mm AM graft was then placed over this area, secured with Tisseel fibrin glue, and sutured on all sides with interrupted 7-0 Vicryl sutures. The corneal defect was then addressed by applying a 10 mm x 10 mm AM fixated with Tisseel fibrin glue. A second layer of 18 mm × 15 mm AM was then placed over the first layer and sutured into place with interrupted 9-0 Vicryl sutures in a mattress fashion. A subconjunctival injection of ceftriaxone was administered inferotemporally and the eye was patched and shielded. Post-operative regimen included topical fortified tobramycin 14 mg/ml and topical moxifloxacin 0.5% every 2 h with a taper over six weeks, ciprofloxacin ophthalmic ointment four times a day for four months, oral moxifloxacin 400 mg daily for four weeks, atropine 1% twice daily for six weeks, and topical prednisolone acetate 1% twice daily with a taper over four months.

At post-operative day 1, the visual acuity was light perception, and IOP was 3 mmHg. Exam revealed 2+ conjunctival injection, scleral thinning inferotemporally covered by UA and AM, cornea with a 9 mm × 8 mm epithelial defect covered with double-layered AM in good position, and a hazy view beyond the cornea. At 1 week, visual acuity remained light perception and IOP was 2 mmHg. Examination revealed 2+ conjunctival injection, UA in good position inferotemporally covering the scleral thinning, and a 4 mm × 4.5 mm corneal epithelial defect with ~50% thinning centrally. At 2 weeks post-operatively, the entire ocular surface was fully epithelialized. By 1 month, vision was HM and IOP was 2 mmHg. Examination revealed 2+ conjunctival injection, and the area with UA was well vascularized. The cornea had diffuse edema, a scar paracentrally, and irregular thinning (greatest ~50% loss nasally). At 5 months, examination revealed a white and quiet conjunctiva, well vascularized area previously covered by UA, and similar corneal findings (Fig. 2). The visual acuity reverted back to light perception, and the patient was confirmed to have an afferent pupillary defect. At this point, treatment options including penetrating keratoplasty and Boston Keratoprosthesis were discussed with the patient, however given the poor visual prognosis, the patient preferred observation.

## 3 Discussion

Bacterial etiologies comprise the majority of IS cases, with *Pseudomonas aeruginosa* being the most common infectious agent.^3^ Bacterial invasion is facilitated by proteinases that degrade the basement membrane and cause cell lysis as well as exotoxins that cause stromal necrosis.^11^^,^^12^ Furthermore, host lysosomal enzymes and oxidative substances produced by neutrophils, keratocytes, and epithelial cells may significantly contribute to the destruction caused by *Pseudomonas aeruginosa.*^13^ This infectious assault and resulting inflammation can eventually lead to scleral ischemia, necrosis, and thinning.

In less than 20% of cases of IS, medical therapy alone (including systemic, topical, and subconjunctival antibiotics) results in complete resolution of infection. There are several possible explanations for this relatively low success rate, including the sclera's dense collagen fibers and avascularity. As a result, surgical debridement is often used adjunctively to decrease the microbial load and improved antibiotic delivery.^4^^,^^14^ Early mechanical debridement combined with antimicrobial therapy has been demonstrated to decrease the length of treatment, increase the rates of globe preservation, and improve prognosis for IS.^4^^,^^14^, ^15^, ^16^ Although well-described in the literature as an effective surgical technique for infectious scleritis,^17^ cryotherapy was not utilized in either of these cases due to the severe degree of scleral thinning and concerns that cryotherapy may increase the risk of perforation.

In this case series, mechanical debridement, UA and AM grafting, and antimicrobial therapy successfully preserved the globe in two cases of necrotizing IS and scleral melt. Case 1 developed IS after pterygium surgery using mitomycin-C and Case 2 developed IS secondary to an intravitreal injection for presumed keratitis-associated endophthalmitis. Scleral melting and IS are well reported complications following pterygium surgery.^5^, ^6^, ^7^ In one series including three cases of *Pseudomonas*-associated keratoscleritis occurring after pterygium excision, early debridement, topical and systemic antibiotics, and low-dose oral prednisone resulted in successful outcomes.^18^ On the other hand, there has only been one prior report of necrotizing IS following intravitreal injection,^19^ but several reports of IS following sub-Tenon injection.^20^, ^21^, ^22^ This highlights the fact that although rare, ocular injection can result in IS and severe complications such as scleral melt. Case 1 had visual recovery following cataract surgery, whereas Case 2 did not regain functional vision following our surgical treatment; this latter case had an afferent pupillary defect along with presenting visual acuity worse than 20/200 vision and endophthalmitis, which are known poor prognostic indicators in IS.^5^

Various tissues and materials have been used to cover the sclera following debridement of large areas.^23^ While AM transplantation has been employed in the treatment of IS, most reports involve the use of various tissue grafts in conjunction with AM. Zheng et al.^24^ successfully used fascia lata grafts in conjunction with AM following surgical debridement for IS, and Siatiri et al.^25^ restored globe integrity and treated inflammation in a case of necrotizing IS caused by *Pseudomonas aeruginosa* using debridement, scleral graft, tenonplasty, and AM transplantation. Finger et al. described the use of super-thick amniotic membrane grafts for ocular surface reconstruction after resection of conjunctival melanoma, squamous cell carcinoma, sebaceous carcinoma, and atypical pterygium, and for scleral necrosis; retention and complete epithelialization were achieved in all cases.^10^ In the present case series, a thick layer of UA was used to cover the sclera which was then covered with thinner AM.

Anatomically, the UA contains the AM as its outer layer and a loose, proteoglycan-rich Wharton's jelly as its inner layer. Both the UA and AM contain collagen, fibronectin, glycosaminoglycans, and HC-HA/PTX3, however UA is about 10 times thicker than AM alone.^26^, ^27^, ^28^ The UA is advantageous compared to other tissue and synthetic materials (such as sclera, cornea, dermis, and autologous fascia lata) because it is readily available off-the-shelf, is relatively transparent for good cosmetic outcomes, is relatively thick and flexible for secure handling, suturability, and filling voids, has low immunogenicity, and has anti-inflammatory, anti-scarring, and pro-regenerative properties. Hence, UA may prevent the ongoing necrotic process by reducing infiltration and inducing apoptosis of activated neutrophils^29^^,^^30^ and macrophages.^31^, ^32^, ^33^ Additionally, one key biological modulator present in both UA and AM, HC-HA/PTX3, upregulates IL-10, downregulates IL-12, and polarizes macrophages toward anti-inflammatory phenotype, all of which play an integral role in the healing process.^26^, ^27^, ^28^ Moreover, the UA used in our cases was well vascularized within 1 month of surgery, a feature which prevents infection and further thinning. Such an effect has also been shown in cutaneous wounds.^34^, ^35^, ^36^, ^37^, ^38^ Together these conditions may provide an optimal healing environment and facilitate recovery of an inflamed ocular surface as shown in these cases.

## 4 Conclusions

This case series highlights the utilization of UA to maintain the scleral integrity and, along with AM transplantation and antibiotics, preserve the globe in cases of severe IS.

## Patient consent

Written consent to publish deidentified medical information and clinical photographs was obtained from the patients. This report does not contain any personal information that could lead to the identification of the patients.

## Funding

No funding or grant support.

## Authorship

All authors attest that they meet the current ICMJE criteria for Authorship.

## Declaration of competing interest

CJR: Consulting: Avedro, Bio-Tissue, Kala, Sun Ophthalmics, Shire.

The following author has no financial disclosures: ZAS.
